# An Integrated View of Stressors as Causative Agents in OA Pathogenesis

**DOI:** 10.3390/biom13050721

**Published:** 2023-04-22

**Authors:** Joseph S. Floramo, Vladimir Molchanov, Huadie Liu, Ye Liu, Sonya E. L. Craig, Tao Yang

**Affiliations:** Laboratory of Skeletal Biology, Department of Cell Biology, Van Andel Institute, 333 Bostwick Ave NE, Grand Rapids, MI 49503, USA

**Keywords:** mechanical overloading, oxidative stress, DNA damage, proteostatic stress, metabolic stress, inflammation, chondrocyte, signaling pathway

## Abstract

Cells in the body are exposed to dynamic external and internal environments, many of which cause cell damage. The cell’s response to this damage, broadly called the stress response, is meant to promote survival and repair or remove damage. However, not all damage can be repaired, and sometimes, even worse, the stress response can overtax the system itself, further aggravating homeostasis and leading to its loss. Aging phenotypes are considered a manifestation of accumulated cellular damage and defective repair. This is particularly apparent in the primary cell type of the articular joint, the articular chondrocytes. Articular chondrocytes are constantly facing the challenge of stressors, including mechanical overloading, oxidation, DNA damage, proteostatic stress, and metabolic imbalance. The consequence of the accumulation of stress on articular chondrocytes is aberrant mitogenesis and differentiation, defective extracellular matrix production and turnover, cellular senescence, and cell death. The most severe form of stress-induced chondrocyte dysfunction in the joints is osteoarthritis (OA). Here, we summarize studies on the cellular effects of stressors on articular chondrocytes and demonstrate that the molecular effectors of the stress pathways connect to amplify articular joint dysfunction and OA development.

## 1. Introduction

**Figure 1:**
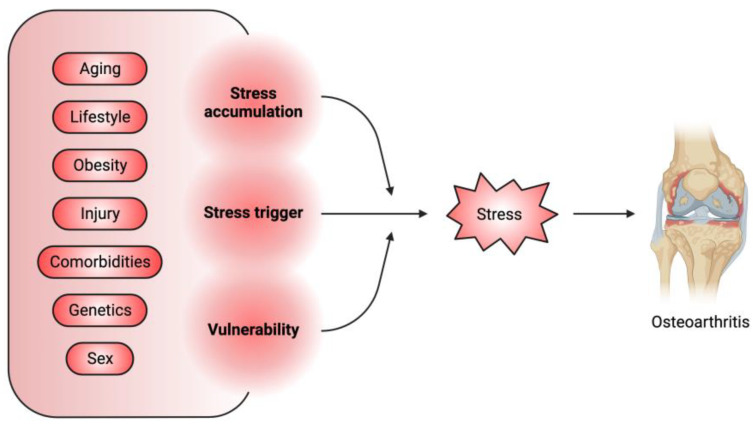
Stress is a major mediator between associated OA risk factors and pathogenesis. The OA-associated risk factors can serve as the initiator of stress or can promote the accumulation of stress-induced cellular damage or aggravate the vulnerability of joint tissues to these damages.

Osteoarthritis (OA) is characterized by the degradation of joint cartilage tissue, chronic local inflammation, and bone remodeling, which result in joint pain, stiffness, swelling, and restricted motion [1,2], with an increased prevalence of over 110% in the last 30 years [3]. OA is highly associated with age, metabolic condition, genetic predisposition, and a history of joint injury or overuse [1]. OA is currently incurable, and standard treatments are pain control or joint replacement [4]. The difficulty in OA treatment reflects the unique properties of the joint tissues, particularly the articular cartilage, which is composed of articular chondrocytes (ACs) and lacks blood and lymphatic vessels [5]. ACs produce and remodel the extracellular matrix (ECM) to maintain a smooth and elastic gliding surface to facilitate movement of and resistance to shocks to the joints [5]. Except for those ACs with progenitor-like properties located in the superficial layer of the articular cartilage, the majority of ACs are long-lived postmitotic cells that are rarely replaced [6]. Over time, ACs accumulate stress-related damage, mostly resulting from mechanical overloading [7], oxidative stress [8], DNA damage, proteostatic stress, and metabolic imbalance. These stressors trigger AC homeostatic defects, including abnormal chondrocyte differentiation [9], senescence and apoptosis [8], a decline in ECM production [10] and an increase in ECM degradation and widespread inflammation [11], leading to OA. Taken together with the associated risk factors, stress acts as a major mediator between these factors and OA pathogenesis (Figure 1).

The long-lived ACs becoming senescent following the accumulation of cellular damage from stressors is supposed to promote AC survival by activating anti-apoptotic pathways, despite severely compromised functionality. Yet stressed ACs also produce the senescence-associated secretory phenotype (SASP), which consists of inflammatory and catabolic factors, such as tissue degrading enzymes [12], de facto functioning as the “contagious” root source of inflammation and tissue degradation in OA [13]. SASP produced from senescent ACs triggers a cascade of pathogenic events that lead to OA [14], supported by augmented senescence observed in cartilage in post-traumatic OA [15]. Overall, the presence of senescent ACs can act as a key component of a vicious loop of OA-related stressors: they can be both the source of new stressors and the products of others.

A key to preventing OA is to harness what we know from decades of work detailing how stressors damage ACs and lead to OA pathogenesis to reduce those molecular mediators of stress in aging joints. In this review, we summarize and integrate ways in which the five primary stressors connect molecularly to give rise to the profound changes to ACs in OA pathogenesis. While generating this schema has allowed us to evaluate a wide breadth of the field, we had to sacrifice detailed coverage to do so. Several excellent reviews exist that cover OA and individual stressors in general, and the reader is directed to them [2,15,16,17,18].

## 2. Primary Stressors in OA Pathogenesis

### 2.1. Mechanical Overloading

Normally, mechanical loading is necessary for joint health: by promoting cartilage thickness and ECM content, and by accelerating the exchange of fluid between the cartilage matrix and synovial cavity to supply the avascular articular cartilage with nutrients [19]. Cyclic loading also increases aggrecan biosynthesis in highly damaged and inflamed lesions, which can aid in repair [20]. In addition, proper level of mechanical loading is a source of anti-inflammatory processes [21,22]. However, excessive or cumulative mechanical stress, as a result of aging, joint injury, repetitive joint loading, joint misalignment, and obesity, damages joint tissues, in particular articular cartilage. In fact, introducing excessive mechanical stress to the joints is the main way to generate post-traumatic OA mouse models [23]. 

Chondrocytes sense mechanical loading through mechanosensing channels, cilia, integrins, and other elements of the focal adhesion complex, all of which convert loading information into the cell via intracellular signaling pathways. The TRPV4 Ca^2+^ preferred ion channel is a mechanosensitive channel present in ACs which increases the expression of ECM proteases under mechanical loading [24]. Chondrocyte-specific loss of TRPV4 alleviates the age-related OA phenotype in mice, suggesting that TRPV4-mediated mechanotransduction pathway can be a possible therapeutic target to treat aging-associated OA [25]. The mechanosensitive Ca^2+^ channels PIEZO1 and PIEZO2 are robustly expressed in ACs, and they potentiate the mechanically induced Ca^2+^ signals. A PIEZO-blocking peptide reduces chondrocyte apoptosis after mechanical injury in an explanted cartilage model, suggesting that PIEZO-mediated cartilage mechanotransduction may be a factor in OA pathogenesis and attenuating its activity may be a potential therapy for post-traumatic OA [26]. Increased intracellular Ca^2+^ concentration as a result of mechanical loading can trigger mitochondrial reactive oxygen species (ROS) production and cartilage degeneration [27]. Mechanical loading can also deform the cytoskeleton and cause damage to the mitochondria, leading to strain-mediated ROS release [27,28]. In a mouse model of OA, mechanical overloading reduces AC expression of superoxide dismutase 2 (SOD2), an enzyme that clears mitochondrial ROS; this exacerbates excessive mitochondrial superoxide formation and promotes AC apoptosis [29]. AC stressors do not act in isolation, and as is already apparent, mechanical overloading acts directly to increase oxidative stress. Oxidative stress in OA will be further elaborated below. 

Another arm of mechanical overloading that initiates OA development is the stimulation of defective joint repair, which requires the removal of damaged cartilage. This usually involves the interplay of integrins, kinase activations, augmented inflammatory factors, and cartilage catabolic enzymes. For example, mechanical overloading of chondrocytes activates integrin (αVβ3 and αVβ5)-mediated FAK and MAPK pathways to induce expression of tumor necrosis factor-α (TNFα), IL-1β, or NF-κB which results in MMP expression and ECM degradation [30]. In another example, mechanical loading leads to TNFα activation of NF-κB, MAPK, and c-Jun pathways and increases cartilage degrading MMP13 and ADAMT4/5 expression [31], ultimately inducing AC apoptosis [32]. Mechanical overloading-induced IL-1β further upregulates the expression of cartilage catabolic enzymes via MAPK pathways, amplifying inflammation by producing proinflammatory factors including COX-2, iNOS, PGE-2, and IL-6 [32]. 

During injury or overloading-induced cartilage remodeling, previously ECM-sequestered growth factors, such as TGFβ and FGF, are released [33]. TGFβ signaling acts as a double-edged sword in OA development. On the one hand, TGFβ blocks chondrocyte apoptosis and prevents cartilage degradation by promoting the production of cartilage matrix and lubricant while suppressing the expression of cartilage degrading enzymes [34,35,36,37]. TGFβ-SMAD2/3 signaling in ACs also has a protective role in AC degeneration and apoptosis following mechanical loading [38]. On the other hand, excessive mechanical load increases TGFβ activation in subchondral bone area, leading to marrow osteoid islet formation and enhanced angiogenesis, thus accelerating osteophyte formation and OA progression [39]. It was further determined that within subchondral bone, there is an uneven distribution of TGFβ. In areas of high mechanical loading, TGFβ expression is higher, but in areas of low mechanical stress, TGFβ expression is even lower than that in healthy tissue; both of these extremes are detrimental [40]. FGF2 expression appears to be beneficial in the joint, as FGF2 inhibits IL-1-induced aggrecanase production, thus alleviating OA progression [41].

Mechanical loading also alters the sensitivity of cartilage tissues to other signaling pathways. For example, tensile strain increases β-catenin levels and sensitizes WNT3A signaling-activated expression of cartilage catabolic proteases [42]. Moreover, chondrocytes under hydraulic pressure alter their expression of miR223, which promotes NF-κB signaling by suppressing the level of an NF-κB signaling inhibitor, IKKα [43]. As a downstream effector of many inflammatory cytokines, NF-κB signaling stimulates the expression of inflammatory factors as well as cartilage-degrading enzymes [44]. In addition, excessive mechanical loading was determined to activate the expression of Gremlin-1, which stimulates NF-κB signaling and promotes inflammation and oxidative stress in joints [45]. 

### 2.2. Oxidative Stress

The oxidative pathway is activated by mechanical strain, but it also integrates the input from several other stressors. ROS are highly reactive free radicals containing oxygen molecules. ROS are a lethal weapon of phagocytic cells to attack and kill invaded pathogens and cancer cells. However, they also oxidize and damage proteins, DNA, and lipids [46]. Cells have an antioxidant system to scavenge ROS, primarily comprised of enzymes such as SOD and catalase, and small molecules, such as glutathione (GSH) and vitamin C [47,48]. Reduced expression of SIRT4, a deacetylase, was detected in OA cartilage; it leads to a decrease in SOD1, SOD2, and CAT expression, building up a more severe oxidative environment [49]. ROS production and oxidative stress are elevated in patients with OA, while OA cartilage has reduced expression of antioxidant enzymes. Together, these lead to an oxidative environment in OA tissue [50]. A major source of ROS is the mitochondrial electron transport chains, out of which 2–3% of O_2_ turns into O_2_^−^. O_2_^−^ is also generated by NADPH oxidase, which is expressed in chondrocytes and also contributes to the OA-promoting oxidative environment that advances OA progression [51,52,53]. Finally, nitric oxide synthase (NOS) catalyzes the reaction that converts arginine into citrulline which produces the free radical, NO^−^ [54]. Inducible NOS (iNOS) is upregulated in ACs by shear stress and proinflammatory factors, including IL-1β, NF-κB, and AP-1 [55]. iNOS loss in ACs prevents OA progression in mouse models, demonstrating that ROS production is a key factor in OA pathogenesis [56,57]. 

One major cellular consequence of oxidative stress is AC apoptosis. Given that ACs are the main cells responsible for cartilage matrix renewal and remodeling and that they are rarely replenished, their death fundamentally undermines the joint. NO is excessively produced from articular cartilage explants undergoing mechanical stress and is a potent inducer of chondrocyte apoptosis [58,59,60]. As a primary donor of ROS, mitochondria are also a victim of ROS and play a key role in oxidative stress-induced OA pathogenesis. ROS, if not cleared in time, can interrupt mitochondrial respiratory chain, reduce ATP production, and mutate mitochondrial DNA (mtDNA) [61,62,63]. H_2_O_2_ production and hyperoxidation of peroxiredoxin suppresses normal redox signaling, leading to an accumulation of H_2_O_2_ and disruption of physiological signaling. OA severity can be reduced in mice with increased expression of the antioxidant mitochondrial catalase [64]. While there is no significant change in expression of peroxiredoxin antioxidants in human ACs from older patients, the ACs are still more prone to hyperoxidation in comparison to ACs from younger patient tissue [65]. 

A recent study identified that oxidative stress-induced mitochondrial damage promotes mitochondrial double-strand RNA (mt-dsRNA) efflux. Chondrocytes exposed to H_2_O_2_, doxorubicin, or acute ionizing radiation (IR) induce cell senescence and elevate the cytosolic level of mt-dsRNAs. This further upregulates the expression of senescence-associated secretory phenotype (SASP), interferon β (IFN-β) and IFN-stimulated genes (ISGs), leading to inflammation through the activation of protein kinase R (PKR) and Toll-like receptor 3 (TLR3) pathways [66]. In addition, elevated levels of mt-dsRNA were detected in the synovial fluid of OA patients and cartilage of post-traumatic OA mice. In addition, removal of mt-dsRNA protects chondrocytes from those stresses, representing a promising strategy to treat OA [66].

Cartilage matrix synthesis is also severely affected in oxidative stress-induced OA progression. Endogenously produced ROS suppresses proteoglycan production in human articular cartilage [64]. In addition, introduction of oxidative stress in healthy donor ankle cartilage steers the normally pro-matrix-synthesis IGF1-PI3K/AKT pathway toward an anti-matrix-breakdown IGF1-MEK/ERK pathway [67]. Higher basal level of the IGF1-MEK/ERK pathway is also observed in human OA chondrocytes [67]. Moreover, inflammatory cytokines, such as IL-1, elevate the level of radicals [68], which further block the synthesis of proteoglycan, collagen type II, and aggrecan, as well as suppress chondrocyte progenitor migration to and proliferation at the injured foci [69].

### 2.3. DNA Damage

DNA double-strand breaks and G/T or G/A transversions occur as a result of free radical oxidization of nucleoside bases [70], irradiation, genotoxins, and/or proinflammatory cytokines, leading to apoptosis and cellular senescence [71,72]. DNA damage increases linearly with age in human chondrocytes [73]. Furthermore, age-matched OA tissue has higher levels of DNA damage in comparison to healthy controls [73]. Elevated oxidative DNA damage in cartilage is positively correlated with OA symptom severity in a porcine OA model [74], and human cartilage explants irradiated to induce double-strand breaks and stimulated with exogenous mitogens, TGFβ and FGF [75], had accelerated chondrocyte senescence. 

Another study reported that the inflammatory factors, IL-1β and TNFα, increase free radicals in human chondrocytes, resulting in mitochondrial DNA (mtDNA) damage, impaired ATP production, and apoptosis [76]. In an additional study using porcine articular cartilage, mtDNA changes were more severe in the OA-associated chondrocytes than in healthy ACs, indicating that the OA-associated ACs are more sensitive to inflammatory signals in producing free radical production [77]. 

Disruption of metabolic pathways, such as the selenium pathway, by depleting selenophosphate synthetase 1 (SEPHS1) increases ROS levels and leads to DNA damage [78]. The *Sephs1*-deficient cells have reduced expression of those stress-related selenoproteins that act as oxidoreductases (including glutathione peroxidase 1 (GPX1) and methionine sulfoxide reductase B1 (MSRB1)) [79]. These deficient cells have stronger staining for γ-H2A.X (a marker of DNA damage) and higher expression of cartilage-degrading proteases ADAMTS5 and MMP13. In addition, *SEPHS1* expression is decreased in human OA transcriptomes. These overall suggest that the selenium metabolic pathway is crucial for maintaining the health of ACs by decreasing ROS, and its loss/deficiency contributes to the progression of OA pathogenesis [79].

Expression of genes involved in DNA damage repair is changed in OA tissue. For example, the excision repair cross-complementation group 1 (ERCC1), an endonuclease required for DNA damage repair, is reduced in OA cartilage [80]. ERCC1 loss increases Mmp13 and suppresses Col2 expression, leading to degradation of ECM, while also promoting chondrocyte apoptosis and senescence [80]. In addition, the expression of cyclic GMP-AMP synthase (cGAS)-stimulator of interferon genes (STING), a component of the innate immune pathway that senses cytosolic DNA fragments derived from DNA damage, is markedly increased in OA tissues or IL-1β treated chondrocytes [81]. Gain of STING function suppresses ECM production, increases the expression of cartilage-degrading enzymes, promotes NF-κB signaling, and leads to chondrocyte apoptosis and senescence. Another recent work reported that irradiation-induced DNA damage stress synergizes with mitogenic stimuli by TGFβ and basic FGF to accelerate chondrocyte senescence in horse and human cartilage explants [75].

DNA damage impacts the expression of nuclear receptors in OA tissue. Estrogen receptor-α (ERα) decreases in human and mouse chondrocytes under Doxorubicin treatment-induced DNA damage [82]. In human OA tissues, the severely damaged regions have increased DNA damage in chondrocytes coupled with decreased ERα expression. Overexpression of ERα partially rescues the senescent phenotype of Doxorubicin-pretreated human ACs via suppressing NF-κB pathway [82]. This suggests that an estrogen-independent mechanism that regulates ERα is needed to maintain AC homeostasis [82,83].

### 2.4. Proteostatic Stress

Proteostasis is the homeostatic state of a functional proteome. It is maintained by the proteostatic network that integrates protein synthesis, folding, trafficking and degradation in various cellular compartments [84]. Chondrocytes, as dedicated secretory cells, have robust protein synthesis activity and large endoplasmic reticula (ERs), so they are particularly vulnerable to proteostatic stress [16]. ER stress, a main type of proteostatic stress, occurs when the capacity for nascent protein folding in the ER becomes impaired. This is usually caused by protein overexpression, expression of misfolding-prone mutant proteins and dysregulated protein trafficking and degradation [16,85]. To handle ER stress, eukaryotic cells have evolved a conserved unfolded protein response (UPR), which relies on three unfolding protein sensing pathways: (1) IRE1/XBP1 pro-survival pathway; (2) ATF4 protective pathway; and (3) the PERK/ATF6/CHOP pro-apoptosis pathway. IRE1 is a transmembrane protein with both kinase and RNase activity. It can sense protein misfolding in the ER to become activated, then generates an XBP1 splicing variant, that encodes a stable form of the transcription factor XBP1 to promote the expression of UPR target genes. ER stress triggers ATF6 translocation from the ER to the Golgi apparatus, where ATF6 is cleaved and activated by S1P/S2P proteases. Cleaved ATF4 also enters the nucleus to activate UPR gene transcription. PERK, a serine/threonine ER protein kinase, phosphorylates and activates eIF2a to promote ATF4 mRNA translation. ATF4 induces the expression of a pro-apoptotic transcription factor, CHOP, leading to apoptosis of damaged cells [86]. 

Chondrocytes are sensitive to ER stress [87], and evidence of ER stress is present in human OA chondrocytes [87,88]. ER stress reduces transcription of cartilage matrix genes and promotes rat chondrocyte apoptosis [89]. Moreover, ER stress increases cartilage degradation via the expression of MMP13 in human chondrocytes [90], and reduces the XBP1-dependent protective UPR while enhancing the CHOP-dependent pro-apoptotic UPR, thus causing chondrocyte death [91]. 

The guiding of nascent protein folding or the degradation of misfolded proteins by chaperones is essential for minimizing protein aggregation and maintaining proteostasis. If the production or function of the molecular chaperones is impaired, accumulating misfolded proteins cause ER stress and induce UPR [84]. It has been observed that the expression of molecular chaperones, such as HSPA5 and BIP, is reduced in aging cynomolgus monkey articular cartilage, which sensitizes chondrocytes to ER stress, and ultimately leads to cell death [92]. In addition, siRNA knockdown of the chaperone, calnexin, in human chondrocytes increases the ER stress markers P-IRE1αm, XBP1, ATF4, and CHOP [92]. 

ER stress can also be induced by metabolic stress. For example, OA chondrocytes from diabetic OA patient cartilage and cultured in the presence of long-term high glucose diet express higher levels of the ER stress makers, GADD34, GRP78, and MANF, but suppress Col2 expression and cell proliferation [93]. In addition, mice fed with high-fat diet develop OA-like lesions exhibiting chondrocyte apoptosis. Treatment with 4-phenyl butyric acid, a chemical chaperone known to ease ER stress, alleviates the OA phenotypes in the high-fat-diet-fed mice [94]. These studies demonstrate that ER stress contributes to high-fat diet or obesity-induced OA, which can be targeted for OA therapeutics.

### 2.5. Metabolic Stress

Metabolites are the source of energy and the building blocks of biomaterials for tissue homeostasis and renewal. However, chronic metabolite imbalance, or improper function in processing or using these metabolites, can bring forth stress to cells and ultimately lead to disease. High dietary fat consumption [95] and type 2 diabetes [95] can both accelerate the progression of knee OA. A meta-analysis integrating multiple studies showed that the risk for knee OA is increased by metabolic syndromes (MetS), defined as the presence of any three of the following components: abdominal obesity, hypertriglyceridemia, low high-density lipoprotein cholesterol; high blood pressure, and high fasting glucose [96]. These metabolic conditions accompany or induce systemic elevation of ROS and proinflammatory cytokines in OA [97]. A connection between high-fat diet, inflammation, and OA development has been established [98]. 

A number of nuclear receptors that regulate lipid metabolism contribute to OA progression by altering the production of cartilage ECM, cartilage degrading enzymes, antioxidants, and oxidative stress inducers [99]. In the case of high-fat diet, palmitate induces IL-1β-stimulated chondrocyte apoptosis [100]. Palmitate also upregulates COX2 and IL6 expression via TLR4 signaling and causes cartilage breakdown [100]. In addition, excessive free fatty acids released from lipid droplets can accumulate and induce apoptosis or metabolic disruption in ACs, defined as lipotoxicity [101]. Lipotoxicity and the pathological phenotypes of OA chondrocytes can be restrained by the protein kinase casein kinase 2 (PKCK2)-six-transmembrane protein of prostate 2 (STAMP2) and fat-specific protein 27 (FSP27) axis, which sequesters free fatty acids [101]. In contrast, a scarcity of lipids determines the chondrogenic fate of skeletal progenitors by activating the forkhead box O (FOXO) transcription factor, which activates *SOX9* gene expression [102]. Besides its well-established function in initiating chondrogenesis, SOX9, in turn, suppresses oxidation of fatty acids, allowing chondrocytes to sustain an avascular microenvironment with low lipid supply [102]. 

Aberrant carbohydrate metabolism, which can be a result of alterations in dietary input, transport, and receptor usage, is also associated with pathological changes in chondrocytes. High-sucrose diet can recapitulate characteristics of early-stage OA, including chondrocyte hypertrophy and higher synovial cellularity independent of weight [103]. The high-sucrose diet also decreases antioxidant proteins, glycolysis-related enzymes, and the expression of genes related to mitochondria function [103]. Glucose is also a major energy source for chondrocytes, and its cellular entry requires the glucose transporter GLUT1. *Glut1* deletion during development disrupts chondrocyte proliferation and hypertrophy resulting in a skeletal phenotype [104]. Notably, *Glut1* expression is mediated by the BMP-mTORC1-HIF1α axis [104]. Extrapolating from the developmental context may provide new insights into AC homeostasis because related signaling pathways are found in OA pathogenesis [105].

Hyperglycemia and aging promote the accumulation of advanced glycation end products (AGE) as a result of a spontaneous reaction that adds a sugar group onto lipids, proteins, and ECM. AGEs trigger signaling via receptor for AGE (RAGE), which increases ROS and inflammation in articular joint tissues and promotes OA development [106,107]. Furthermore, the accumulation of intracellular AGEs induces ER stress and leads to CHOP-mediated chondrocyte apoptosis [108].

## 3. An Integrated Schema of Stressors in OA and Joint Aging

**Figure 2:**
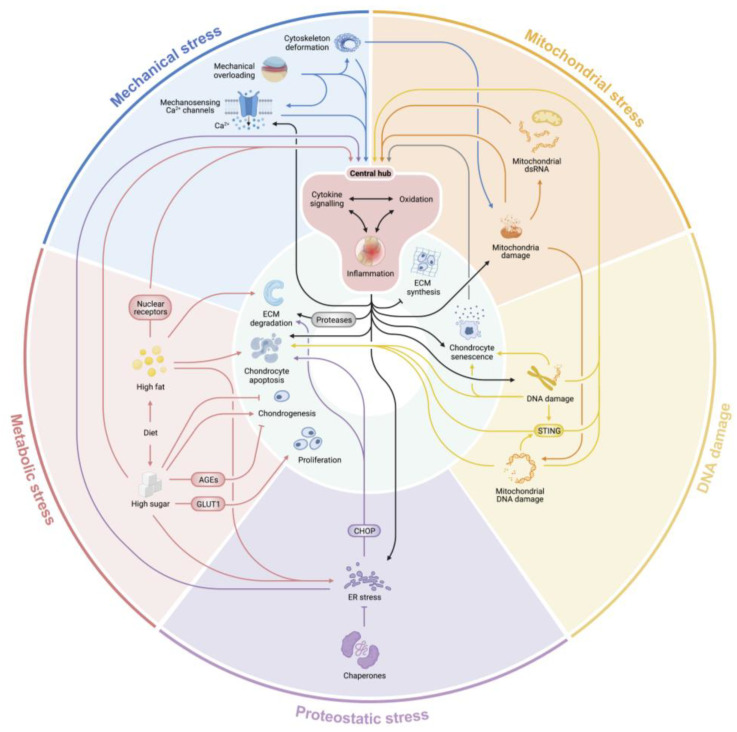
An integrated view of the molecular synergy among stressors that leads to articular chondrocyte dysfunction and OA pathogenesis. The inner circle represents the hallmarks of OA, including chondrocyte apoptosis, senescence, decline in ECM production, increase in ECM degradation, abnormal chondrocyte differentiation, and inflammation. These OA hallmarks are the consequences of the five major stressors placed in the outer circle. The arrows indicate the interplay of these stressors and their downstream effectors. Note that the oxidative and proinflammatory factors form a central hub (in red) integrating the output of all stressors and aggravating the stress-induced damage with multiple vicious feed-forward loops.

With age, cellular damages induced by these five primary stressors accumulate in ACs, and in conjunction with the diminution of anti-stress mechanisms, they generate a hostile environment for AC homeostasis [109]. These ultimately lead to the manifestation of aging phenotypes of joints or pathology of OA by promoting the hallmarks of AC defects: abnormal chondrocyte proliferation and differentiation, chondrocyte senescence and apoptosis, cartilage matrix loss (caused by either insufficient matrix production or overactive matrix-degrading enzymes), and inflammation (Figure 2). 

Those stressors do not act alone; instead, extensive feed-forward loops and cross-talk between them snowball to accelerate AC changes that give rise to OA progression over time. For example, mechanical stress causes oxidative stress, inflammation, and signaling changes. Inflammatory signaling sensitizes Piezo1 response to mechanotransduction in articular chondrocytes as a pathogenic feed-forward mechanism in osteoarthritis [110]. Oxidative stress can further induce DNA damage and mitochondrial dysfunction, which leads to mt-dsRNA efflux, cell senescence, apoptosis, and inflammation. Cell senescence causes the release of inflammatory factors and matrix-degrading enzymes, while in turn, inflammation aggravates oxidation, DNA damage, and senescence, forming a vicious loop advancing cartilage damage. Yet this interconnectedness also means that alleviating one stressor can improve the damage and symptomology caused by the others. For example, oxidation-induced cartilage abnormality can be ameliorated by ER stress inhibitors, while antioxidants can reciprocally alleviate ER stress-induced OA [94,111]. 

It is worthy of note that oxidative stress and inflammation are the two nexuses with the most connections to other stressors and AC defects, suggesting that they serve as a central hub to integrate the actions of these stressors as the main path impacting OA pathogenesis (Figure 2) and can thus be effective targets for OA treatments. Indeed, antioxidants and anti-inflammatory medications show great promise for OA treatment [111,112]. Anti-inflammatory drugs are widely used to mitigate OA-related pain [113]. In addition, Helper-dependent adenovirus (HDAd)- or adeno-associated virus (AAV)-delivered IL1R antagonist (IL1Ra) showed a plausible effectiveness in reducing OA symptoms in small and large animal models [114,115,116]. In recent years, compounds that target the oxidative stress pathways have been extensively explored as possible therapeutic options; these include a number of natural compounds (nutraceuticals), particularly plant polyphenols and flavonoids, such as the extracts of Sida tuberculate or green tea, quercetin, curcumin, and naringenin. They showed promising anti-oxidative and anti-inflammatory effects in the in vivo and ex vivo OA models [111,112,117,118,119]. In addition, other antioxidative agents, such as manganese dioxide nanoparticles, scavenge ROS and exhibit protective effects against cartilage degeneration [120]. Of note, targeting the central hub can alleviate symptoms and prevent progression of OA effectively, but they need to be used continuously if the root causes of oxidation or inflammation are not finally removed. As an example, senescent chondrocytes are the propagators of inflammation, so anti-inflammatory treatments are largely palliative rather than curative. Recently, senolytic drugs developed to kill senescent cells have been used to treat OA in mouse models with ongoing clinical trials [121]. By removing the root cause of inflammation, senolytics are postulated to be not only therapeutic but curative for OA. Nevertheless, as a complex degenerative disease, OA can be involved with multiple stress-producing root causes, which may be difficult to remove completely. In these scenarios, targeting the key factors in the central hub will likely lead to more cost-effective outcomes.

In past decades, the collective efforts of many groups have contributed to the evolution and refinement of such an integrated view of stressors in OA development. However, we can also see the disproportionate number of studies among various stressors. This may reflect the fact that some stressors contribute more heavily than others, but may also be partially due to our current lack of knowledge or technology in elucidating the involvement/mechanism of certain stressors. In recent years, along with the prosperity of epigenomic and metabolomic technologies, our understanding of the pathogenesis of degenerative diseases and cancers has been greatly deepened. We expect that in the future, new technology, such as those mentioned, can help us uncover more crosstalk/interconnection among these stressors and provide new nexuses to be used as targets for developing fruitful therapeutics for OA. 

## Data Availability

No new data were created or analyzed in this study. Data sharing is not applicable to this article.
